# Wave Propagation in Composites of Plasma and Metamaterials with Negative Permittivity and Permeability

**DOI:** 10.1038/s41598-019-39923-7

**Published:** 2019-02-28

**Authors:** Hyunjun Kim, Jeffrey Hopwood

**Affiliations:** Electrical and Computer Engineering, Tufts University, 161 College Avenue, Medford, Massachusetts 02155 USA

## Abstract

Wave propagation is observed through a negative permeability metamaterial immersed in gaseous plasma. A 3D array of split ring resonators (SRR) is enveloped by an inductively heated argon plasma with a nominal plasma frequency of 2.65 GHz. Transmission spectra show electromagnetic waves traverse the composite medium from 1.3–1.7 GHz for which the permeability of the SRRs and the permittivity of the plasma are simultaneously negative. Only surface waves and evanescence are observed outside this frequency band. The edge of the transmission band also shows negative group velocity, albeit with high wave attenuation. The free electron density of the plasma is coupled to the inductive heating, allowing dynamic reconfiguration of the metamaterial’s frequency band and wave impedance.

## Introduction

Negative index materials (NIMs) exhibit negative effective permittivity ${\varepsilon }_{eff}$ and negative permeability ${\mu }_{eff}$ simultaneously. Also called left handed materials (LHM) or double negative media (DNM), the phenomena^1–3^ is typically observed within a narrow, predetermined frequency band. Veselago^4^ is credited with introducing the concept of NIMs in 1968. A transverse electromagnetic wave propagates with a wave vector $k=\frac{\omega }{c}\sqrt{\varepsilon }\sqrt{\mu }$. If the relative permittivity or permeability is negative, the wave vector becomes imaginary and exponential decay (evanescence) of the wave occurs. However if both *ε* and *μ* are negative, the wave vector becomes real and negative, resulting in a negative phase velocity and a negative index of refraction.

Smith^5^ *et al*. demonstrated NIM behavior using a composite structure (*i*.*e*., a metamaterial) of periodic short wires and split ring resonators (SRR). Many researchers have reported electromagnetic properties based on this configuration^6,7^ for which the ensemble of electric dipoles creates an effective permittivity with ${\varepsilon }_{wire} < 0$ and the magnetic resonance of the SRR gives an effective medium with ${\mu }_{SRR} < 0$. The existence of NIM suggests a wide range of applications^8–10^ including cloaking devices, optical detectors and imagers, and advanced antenna systems.

Gaseous plasma have dynamic fundamental properties that are readily manipulated through their free electron density, ${n}_{avr}$. This has resulted in an interest in the fundamental plasma behaviors which may be usefully coupled with metamaterials. In conventional metamaterials, the electric dipole resonance giving ${\varepsilon }_{eff} < 0$ is analogous to ionized gases for which the real part of the complex permittivity is^11^

1 $${\varepsilon }_{eff}=1-\frac{{e}^{2}{n}_{avr}}{{\varepsilon }_{0}m({\omega }^{2}+{\nu }_{m}^{2})}\approx 1-\frac{{\omega }_{pe}^{2}}{{\omega }^{2}}$$

2 $${\omega }_{pe}={(\frac{{e}^{2}{n}_{avr}}{{\varepsilon }_{0}m})}^{\frac{1}{2}}$$

where ${\omega }_{pe}$ is the effective plasma frequency which depends on ${n}_{avr}$, the free electron density averaged over a volume on the order of ${(\lambda /2)}^{3}$, and *e*, *m*, ${\varepsilon }_{0}$, and ${\nu }_{m}$ are the electron charge, electron mass, permittivity of vacuum, and electron-atom collision frequency. As plasma exhibits variable electromagnetic response, it is possible to modulate^12^ EM waves. According to the approximation in Eqn. (1), the collisionless plasma (${\nu }_{m}\ll \omega$) exhibits effective negative permittivity for $\omega  < {\omega }_{pe}$ and this phenomena is promising for reconfigurable^13^ NIMs.

Recently a composite structure of three dimensional periodic plasmas aligned with SRRs demonstrated that plasma damps the magnetic resonance of the SRR such that ${\mu }_{SRR} > 0$ if the conductive rings are not carefully isolated^14^ from the plasma. In addition, the local plasma permittivity (${\varepsilon }_{p} < 0$) reduces the permittivity of the SRR environs causing an upward shift^15^ in the negative permeability frequency band. Here we report plasma characteristics and SRR metamaterial configurations that overcome those limitations^14^ that have prevented NIM behavior. Specifically, previous reports of NIM transmission through plasma metamaterials have overlooked the quenching and frequency shifting of SRR resonance. In addition, the absence of broadband transmission spectra in these reports calls into question the physical origin of transient transmissions. This work explores the nature of transmission through the metamaterial due to surface waves versus electromagnetic waves, and then defines the conditions necessary for NIM behavior.

## Methods

**Figure 1:**
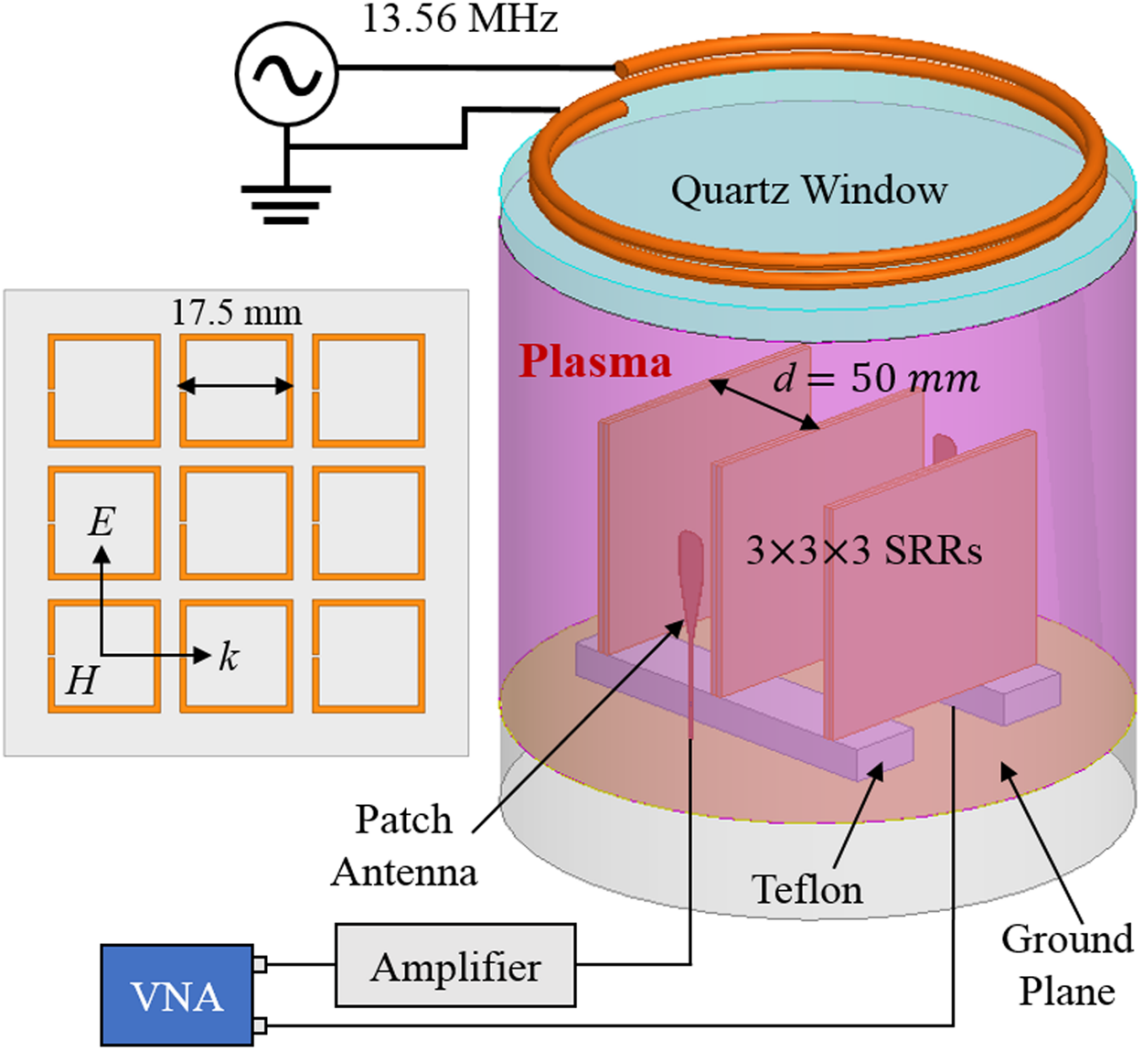
Experimental setup showing split-ring resonators (SRRs) within the plasma volume. Transmission is determined by a vector network analyzer (VNA) and a pair of patch antennas.

We experimentally verify the electromagnetic response of plasma-reconfigured NIMs by direct measurement of wave transmission spectra (*S*_21_) through a plasma-SRR composite. The negative permeability is due to rectangular SRRs fabricated from copper-clad dielectric substrates as seen in Fig. 1. Each substrate (Rogers^TM^ TMM3, ${\varepsilon }_{r}=\,3.27$, thickness of 1.5 mm) hosts nine SRRs in a (3 × 3 × 1) configuration. The 3D metamaterial consists of an ensemble of adjacent substrates such as the (3 × 3 × 3) array in Fig. 1. Critically, the SRRs are isolated from plasma damping by capping each substrate with an identical “blank” TMM3 dielectric layer.

The SRR array is placed above a copper ground plane using Teflon supports $({\varepsilon }_{r}=\,2)$. A vertically polarized electric field is generated by a small patch antenna positioned in front of the metamaterial and an identical antenna is positioned to the rear of the array. A vector network analyzer (VNA) determines the magnitude, phase, and group delay through the metamaterial. An amplifier placed between the output port of the VNA and the front antenna amplifies the probe signal (1–3 GHz) to 1 W resulting in improved signal to noise performance. The two patch antennas near the ends of the SRR array are covered by Teflon cylinders (not shown) to prevent contact with the plasma.

Plasma is introduced within the metamaterial by mounting the copper ground plane to the baseplate of a vacuum chamber. With the metamaterial and baseplate inserted, the vacuum chamber is maintained at an argon pressure of 0.13 Pa (1 mTorr). The plasma is inductively heated at 13.56 MHz through a quartz window located above the SRR array. Heating occurs within a skin depth (~2 cm) of the quartz window^16^ and diffusive ambipolar transport fills the regions surrounding the SRR substrates. The plasma generator, as described in detail elsewhere^17^, creates an electron density *n*_*e*_ of $1-5\times {10}^{11}\,c{m}^{-3}$ using inductive heating power from 100 to 900 W *without* insertion of the SRR array. The electron-argon collision frequency is the order of 10^6^ *s*^−1^ so that the plasma satisfies the collisionless criteria according to Equation (1). In addition, the plasma that surrounds the periphery of the metamaterial prevents wave propagation from the patch antenna to the metal vacuum chamber walls and eliminates reflections and associated chamber resonances for $\omega  < {\omega }_{pe}$.

For calibration of the measured transmission, entirely *blank* TMM3 substrates replace the SRR substrates and the resulting transmission is defined as the 0 dB reference with zero phase. In this sense, *S*_21_ represents the *relative* transmission through the metamaterial compared to the transmission through the system with blank dielectrics. Transmission spectra above 0 dB do not represent gain, but merely signify configurations for which more energy is detected at the receiving antenna compared to the case of blank substrates.

## Results and Discussion

**Figure 2:**
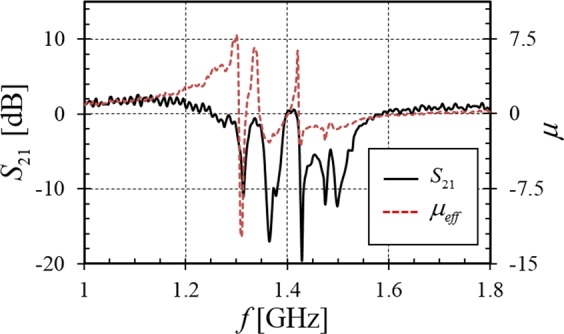
Measured transmission spectra *S*_21_ and extracted effective permeability ${\mu }_{eff}$ of the SRR array measured outside of the plasma chamber.

Figure 2 describes the magnitude of the transmission through the (3 × 3 × 3) SRR metamaterial without plasma present. The non-transmissive frequency band from 1.3–1.6 GHz shows the region for which ${\mu }_{eff} < 0$ and the microwaves are evanescent. This band corresponds to the various modes of the coupled resonators as predicted by coupled mode theory^18^ and is described in detail elsewhere^19^. The effective permeability of the SRR medium is extracted from the magnitude and phase of *S*_21_ using well-known methods^20,21^. The material shows bands of negative permeability that correlate with the ensemble resonances of the SRRs. Note that there are interspersed bands of positive and negative permeability which are important to later discussions.

**Figure 3:**
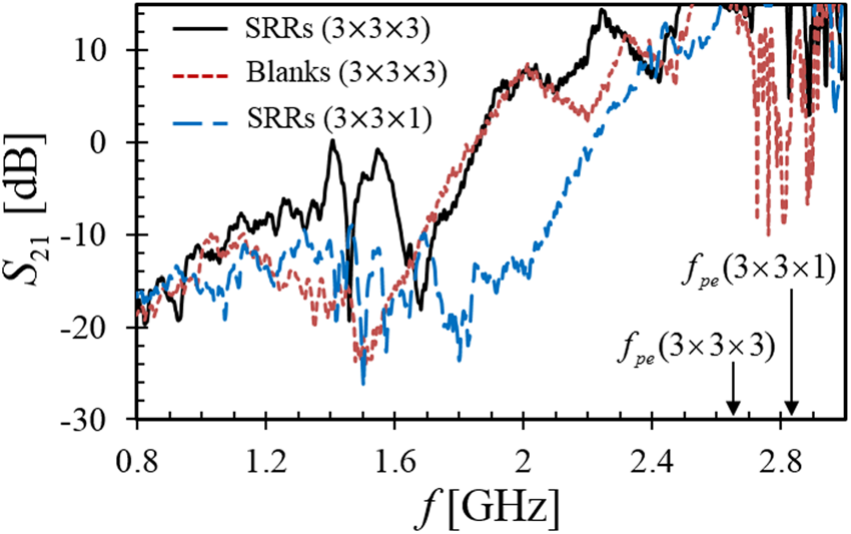
Measured transmission spectra *S*_21_ of SRRs (3 × 3 × 3), blanks (3 × 3 × 3), and subwavelength SRRs (3 × 3 × 1) using an inductive plasma heating power of 500 W. The calculated plasma frequency (*f*_*pe*_) is labeled for the (3 × 3 × 3) and (3 × 3 × 1) SRR configurations.

Figure 3 shows the measured relative transmission magnitude (*S*_21_) for three cases. The first case is the control experiment consisting of three blank TMM3 substrates immersed in the plasma. In this case we observe classic plasma wave transmission behavior. At lower frequencies the electromagnetic (EM) wave is evanescent - due to negative plasma permittivity when $\omega  < {\omega }_{pe}$ - and the measured transmission is −20 dB less than through free space. The transition toward EM wave transmission occurs gradually between 1.7 and 2.65 GHz because the plasma is heated from the top, resulting in an electron density gradient. Above 2.65 GHz the EM wave propagates freely and several cavity resonances within the plasma-filled vacuum chamber are observed near 2.8 GHz where $\omega  > {\omega }_{pe}$. These resonances are numerically modeled as a function of electron density, compared with the experimental observation, and then used to determine the plasma frequency. Above the plasma frequency we note that *S*_21_ is greater than 0 dB because the microwave energy is partially trapped in the plasma chamber and transmission is enhanced relative to the free-space calibration. From the estimated plasma frequency, we find that the plasma surrounding the dielectric substrates has ${n}_{avr}\approx 8.7\times {10}^{10}\,c{m}^{-3}$ and ${\varepsilon }_{eff}\approx -2$ within the SRR resonance frequency band. We note that the collisionless plasma frequency (2.65 GHz) is greater than the frequency bands of negative permeability shown in Fig. 2 such that the plasma establishes the desired negative permittivity.

There is some transmission (−10 dB) in the control experiment near 1.2 GHz. This is the frequency for which a surface plasma polariton^22,23^ is expected to propagate along the interface between the plasma and the TMM3 substrate^24^. It is a confounding wave that must be disambiguated from the NIM frequency bands. The cutoff frequency of the polariton is ${\omega }_{c}={\omega }_{pe}/\sqrt{1+{\varepsilon }_{r}}\approx {\omega }_{pe}/2$ for TMM3. Hence, the surface wave cutoff (1.3 GHz) is half the estimated plasma frequency (2.65 GHz) as observed in all spectra reported here. The SRR metamaterial resonance (1.3–1.6 GHz) was purposely chosen to avoid overlapping this surface wave. Finally, we note that there may also exist a propagating surface polariton^25^ along the metal ground plane. This surface wave propagates below the plasma frequency^26^ and likely contributes to the indistinct transition of *S*_21_ near the plasma frequency. The metamaterial’s NIM band was intentionally chosen to reside below this surface wave.

Having established and properly controlled for confounding wave propagation, the (3 × 3 × 3) SRR metamaterial is immersed in the plasma. Referring to Fig. 3, the wave transmission in the band of negative permeability (1.3–1.6 GHz) is shown to be 20 dB higher than the control case and, with two maxima of 0 dB, the NIM transmission is comparable to the free-space system calibration. A narrow band of non-transmission appears in the middle of the NIM region of Fig. 3 that aligns to ${\mu }_{eff} > 0$ in Fig. 2. Similar behavior was observed with (3 × 3 × 4) and (3 × 3 × 5) configurations, but with reduced plasma density and ${\omega }_{pe}$ due to greater surface recombination of plasma electrons on the more closely spaced substrates.

The spacing between TMM substrates is approximately 50 mm as shown in Fig. 1. The relatively large spacing reduces surface recombination losses of plasma electrons and increases the uniformity and density of the plasma within the metamaterial. A second constraint, however, is that an EM wave requires a material width in the direction of the H-field of ${d}_{H}\ge \lambda /2$ to propagate^27^ through the material. Using the relative permittivity and permeability found in the following sections, we calculate that the material must be the order of 60 *mm* wide to avoid dimensional wave cutoff in the NIM. To show that the NIM is supporting an EM wave, we measure the transmission with only one SRR substrate such that ${d}_{H} < 60\,mm$. Figure 3 demonstrates that the NIM transmission band is absent in this narrow (3 × 3 × 1) configuration, suggesting the (3 × 3 × 3) material supports an EM wave and not merely a surface wave or quasistatic coupling along a single SRR array substrate. As a side note, the surface plasma polariton near 1.2 GHz still exists on the single substrate, but its transmission magnitude is reduced due to the absence of two of the TMM3 substrates. Finally, the plasma frequency is 0.2 GHz higher in this narrow configuration (2.85 GHz) because the deleterious surface recombination of free electrons on the outer two TMM3 substrates is eliminated in the (3 × 3 × 1) case.

**Figure 4:**
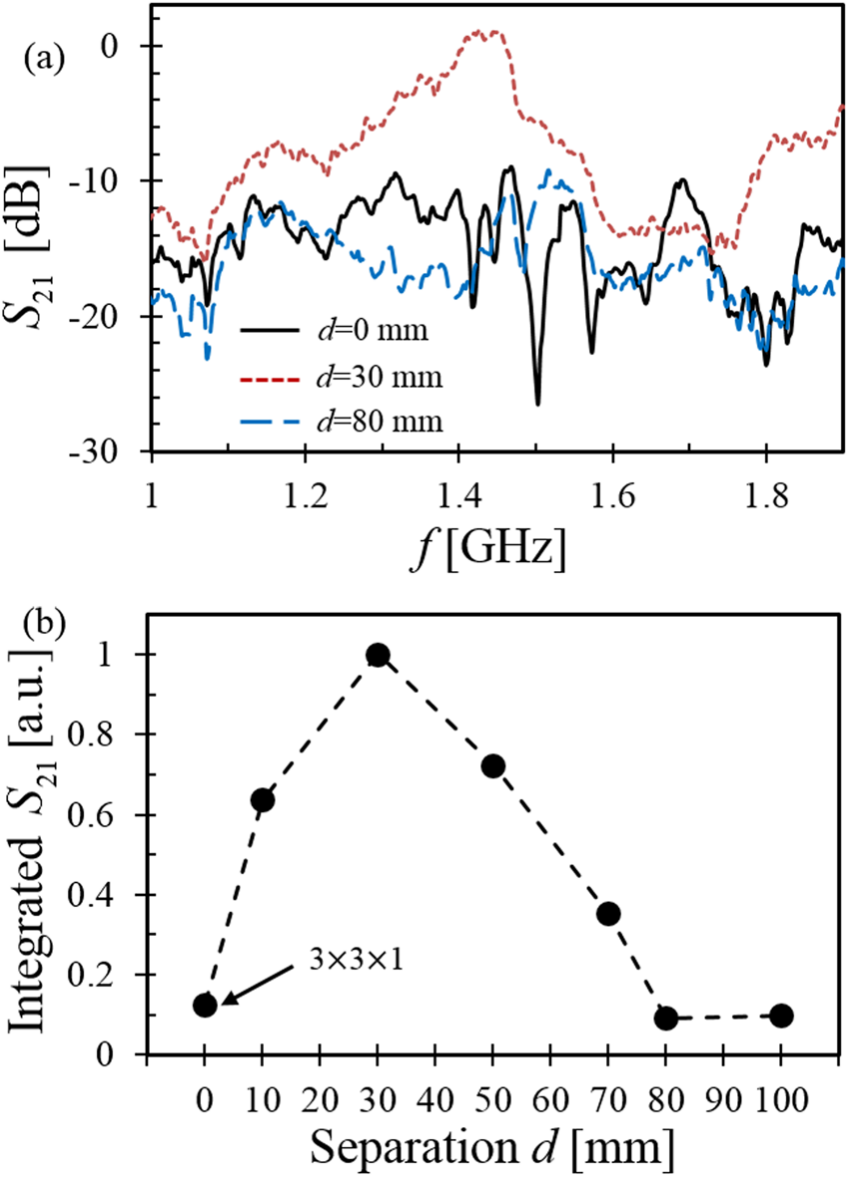
The wave transmission through (3 × 3 × 2) SRRs versus the separation between substrates, *d*, with an inductive plasma heating power of 500 W; (**a**) Measured transmission spectra *S*_21_, (**b**) *S*_21_ integrated over the transmission frequency range of 1.3–1.7 GHz. The (3 × 3 × 1) SRR configuration was used for the *d* = *0* *mm* case.

The two limiting cases described above are investigated more thoroughly by using a (3 × 3 × 2) configuration and systematically recording the transmission spectra while increasing the separation between the two substrates. The sample spectra presented in Fig. 4(a) show very little transmission when *d* = 0, maximum transmission for *d* = 30 mm and a return to minimal transmission as *d* increases to 80 mm. The full data set is summarized in Fig. 4(b) which shows the integrated NIM transmission (from 1.3 to 1.7 GHz) as a function of the separation between the SRR substrates (*d*). Understanding these data requires a knowledge of the extent of the negative permeability region away from the plane of the substrate. If one approximates the SRR as an isolated current loop with radius *b*, the axial magnetostatic field responsible for effective permeability is well-known to be

3 $${B}_{o}=\frac{{\mu }_{o}{I}_{o}{b}^{2}}{2{({z}^{2}+{b}^{2})}^{3/2}}$$

where *I*_*o*_ can also be regarded as the current induced by a non-resonant impressed B field, *B*_*o*_. At resonance, however, the current in the ring is *QI*_*o*_, where *Q* is the quality factor of the resonator. Therefore the resonance magnetic field is a factor of *Q* larger, and in the opposite direction. Using this approximate geometry and finding the point along the axis of the loop at which the total B-field crosses zero (*z*_*o*_) gives an estimate for the extent of the negative permeability:

4 $${z}_{o}\approx {Q}^{1/3}b$$

For the present SRR geometry (2*b*~17.5 mm) and quality factor (*Q*~25–50), we estimate *z*_*o*_ to be 25–30 *mm*. Neglecting substrate thickness, the width of the NIM transmission region is ${d}_{H}=2{z}_{o}+d$. Although wave transmission also depends on impedance matching as described later, a picture emerges from the data of Fig. 4(b): For narrow separations, *d*, the wave is non-transmitting because the NIM is too narrow to support the EM wave. As *d* approaches 30 mm, the transmission channel is fully formed and transmission is maximized. In the (3 × 3 × 2) configuration explored here, we see that if the spacing between boards exceeds the range of negative permeability (*d* > 2*z*_*o*_) there exist regions containing slabs of plasma (*ε*_*eff*_ < 0) with positive permeability such that the observed transmission decreases to the case of a single narrow channel (3 × 3 × 1). This occurs for *d* > 70 *mm* because the pair of substrates is too widely spaced to create a continuous NIM region.

**Figure 5:**
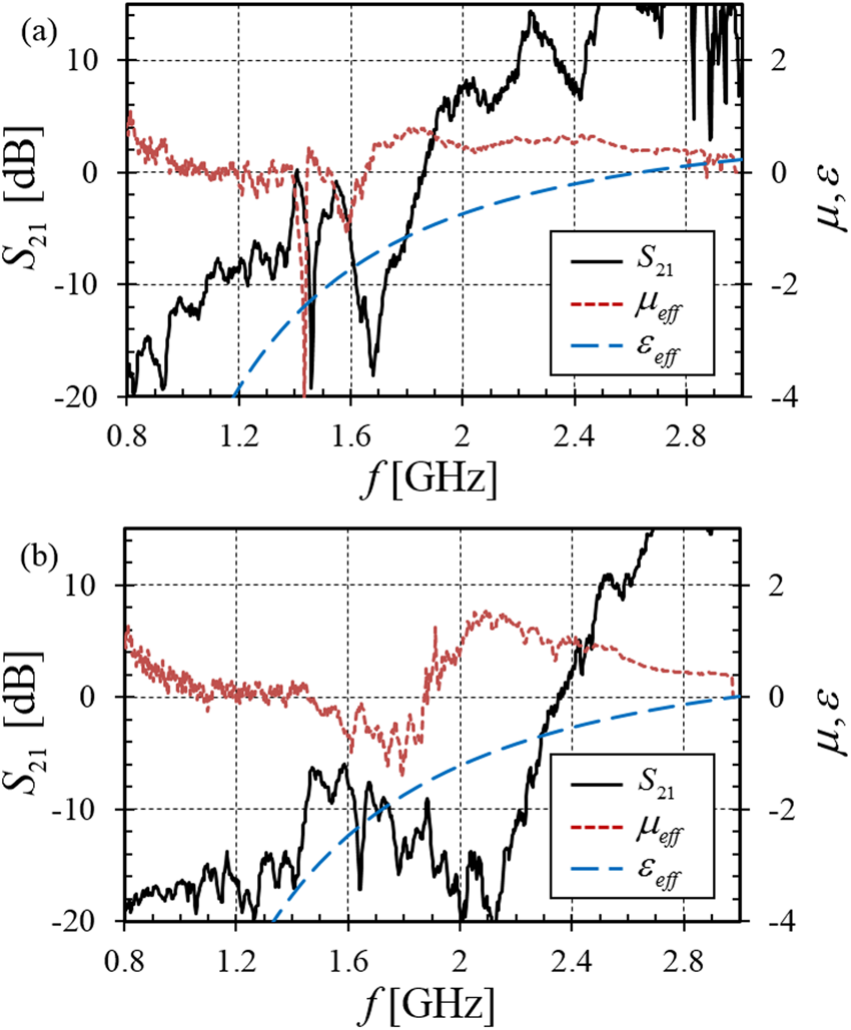
Measured transmission spectra *S*_21_, effective permeability ${\mu }_{eff}$, and effective permittivity ${\varepsilon }_{eff}$ of (3 × 3 × 3) SRRs vs. inductive plasma heating power, (**a**) 500 W, (**b**) 900 W.

The effective permeability of the SRR/plasma composite is extracted^20,21^ from *S*_21_ with the aid of the plasma permittivity in Eqn. (1) as found from the measured plasma frequency. Figure 5 shows the extracted ${\mu }_{eff}$ along with the estimated ${\varepsilon }_{eff}$ and the measured *S*_21_ repeated as a visual reference. The effective permeability is confirmed to be negative in the presence of plasma, although the permeability is observed to be less negative as the plasma density is increased as shown in Fig. 5(b). Note that the minimum magnitude of ${\mu }_{eff}$ without plasma is less than −10, but it decreases with higher plasma density until ${\mu }_{eff}\approx -1$ at 900 W. The proximity of plasma damps the SRR resonance even though the SRR is encapsulated in the dielectric substrates, ultimately resulting in loss of negative permeability at very high electron density. The permeability also becomes positive^14^ if the plasma contacts the SRR. These are critical observations that have been ignored in previous claims of plasma-NIM transmission. One also observes that the NIM frequency band shifts upward as electron density increases because the increasingly negative plasma permittivity diminishes the *net* permittivity of the TMM3 layers and increases the resonant frequency of individual SRRs^14,15^. The NIM transmission is reduced in the case of higher plasma density due to (1) the unavoidable evanescent wave region between the antenna and the metamaterial containing only overdense plasma, and (2) a wave impedance discontinuity between the Teflon antenna shields and the NIM. Ideally, the NIM wave impedance should match Teflon with $\eta =\sqrt{|{\mu }_{eff}|/|{\varepsilon }_{eff}|}=\sqrt{1/2}$ and the transmission is maximized in Fig. 5(a) under this condition. The variation of the transmission frequency band and the NIM wave impedance is readily controlled using the power absorbed by the inductively coupled plasma, demonstrating the possibility of real-time plasma reconfiguration of metamaterials.

**Figure 6:**
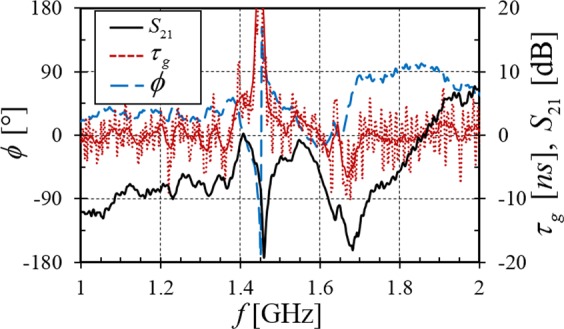
Measured transmission spectrum *S*_21_, group delay ${\tau }_{{\rm{g}}}$, and phase *ϕ*. The solid group delay curve is smoothed using the experimental data shown by the dotted curve.

Figure 6 examines the phase response (*ϕ*) and group delay $({\tau }_{{\rm{g}}})$ of the NIM, repeating the transmission response as a visual reference. The group velocity can be simply determined from the measured phase *ϕ* or group delay ${\tau }_{{\rm{g}}}$ using^28^ the length of the media *L* = 72 mm,

5 $${v}_{{\rm{g}}}=\frac{\partial \omega }{\partial k}=\frac{L}{-\partial \varphi /\partial \omega }=\frac{L}{{\tau }_{{\rm{g}}}}$$

where *k* is the wave vector. Within the transmitting regions with ${\mu }_{eff} < 0$ and ${\varepsilon }_{eff} < 0$ we find positive group velocity as expected, except near 1.65 GHz where the group delay and group velocity become negative. This region of anomalous dispersion is due to the positive slope of the phase and is often observed in conventional NIMs without plasma^28,29^. The negative group velocity phenomena is associated with strong wave attenuation. This is the reason that *S*_21_ is greatly reduced around 1.65 GHz even though the effective permeability remains negative.

In summary, electromagnetic wave propagation exists through a volume of effective negative permeability in the presence of collisionless plasma. Control experiments have isolated the EM wave in the negative index material from plasma-surface waves. The free electron density of the plasma not only creates the requisite negative permittivity, but also couples to the negative permeability medium, dynamically altering its magnetic response ${\mu }_{eff}$ and wave impedance. The plasma/metamaterial composite also exhibits anomalous dispersion and negative group delay.
